# Comprehensive Analysis of the Expression and Prognosis for MMPs in Human Colorectal Cancer

**DOI:** 10.3389/fonc.2021.771099

**Published:** 2021-11-05

**Authors:** Jing Yu, Zhen He, Xiaowen He, Zhanhao Luo, Lei Lian, Baixing Wu, Ping Lan, Haitao Chen

**Affiliations:** ^1^ Department of Colorectal Surgery, The Sixth Affiliated Hospital of Sun Yat-sen University, Guangzhou, China; ^2^ Guangdong Provincial Key Laboratory of Colorectal and Pelvic Floor Diseases, Guangdong Institute of Gastroenterology, Guangzhou, China; ^3^ Guangdong Provincial Key Laboratory of Malignant Tumor Epigenetics and Gene Regulation, Guangdong-Hong Kong Joint Laboratory for Ribose Nucleic Acid (RNA) Medicine, Ribose Nucleic Acid (RNA) Biomedical Institute, Medical Research Center, Sun Yat-sen Memorial Hospital, Sun Yat-sen University, Guangzhou, China; ^4^ School of Public Health (Shenzhen), Sun Yat-sen University, Shenzhen, China; ^5^ School of Public Health, Shenzhen Campus of Sun Yat-sen University, Shenzhen, China

**Keywords:** colorectal cancer, MMPs, prognosis, expression, tumor stage

## Abstract

**Background:**

Previous study implicated that genes of matrix metalloproteinase (MMP) family play an important role in tumor invasion, neoangiogenesis, and metastasis. However, the diverse expression patterns and prognostic values of 24 MMPs in colorectal cancer are yet to be analyzed.

**Methods:**

In this study, by integrating public database and our data, we first investigated the expression levels and protein levels of MMPs in patients with colorectal cancer. Then, by using TCGA and GEO datasets, we evaluated the association of MMPs with clinicopathological parameters and prognosis of colorectal cancer. Finally, by using the cBioPortal online tool, we analyzed the alterations of MMPs and did the network and pathway analyses for MMPs and their nearby genes.

**Results:**

We found that, MMP1, MMP3, MMP7, MMP9–MMP12, and MMP14 were consistently upregulated in public dataset and our samples. Whereas, MMP28 was consistently downregulated in public dataset and our samples. In the clinicopathological analyses, upregulated MMP11, MMP14, MMP16, MMP17, MMP19, and MMP23B were significantly associated with a higher tumor stage. In the survival analyses, upregulated MMP11, MMP14, MMP17, and MMP19 were significantly associated with a shorter progression-free survival (PFS) time and a shorter relapse-free (RFS) time.

**Discussion:**

This study implied that MMP11, MMP14, MMP17, and MMP19 are potential targets of precision therapy for patients with colorectal cancer.

## Introduction

Colorectal cancer (CRC) is the second leading cause of worldwide cancer mortality. It accounts for 9.2% of all cancer deaths according to the Global Cancer Statistics 2020 (1). In the USA, according to the SEER database, those with CRC have an overall 5-year survival rate of ~64%, primarily dependent on pathological stage at diagnosis. CRC patients diagnosed with disease limited to the colon have greater than 90% 5-year survival rate. Five-year survival decreases to ~70% with regional spread, and for patients diagnosed with distant metastases, the 5-year survival rate drops to 12.5% (2). Despite the significant advances in screening and diagnosis, there are limited therapeutic options for patients with advanced disease, which highlight the need for additional tumor molecular markers and prognostic predictors (3).

The human matrix metalloproteinases (MMPs) family belongs to the metzincin superfamily. The main function of MMPs is catalyzing the proteolytic activities and aiding breakdown of the extracellular matrix (ECM) (4). By degrading connective tissue between cells and in the lining of blood vessels, they enable tumor cells to escape from their original location and seed metastases (5). A large body of experimental and clinical evidence has implicated MMPs in tumor invasion, neoangiogenesis, and metastasis (6). Also, from the 1990s to early 2000s, inhibitors of MMPs (MMPI) were studied in various cancer types. However, despite strongly promising preclinical data, all trials failed due to lack of efficacy and severe side effects (7–9). One important reason to explain the failure is that some MMPs have antitumor effects, while the broad-spectrum MMPIs used in the initial trials might block these MMPs and result in tumor progression (10). Recently, with growing knowledge of MMPs in tumor invasion and metastasis and broader roles in cancer biology, narrow-spectrum MMPIs which were safer and more selective were currently being developed (11).

MMPs play complex and distinct roles in CRC. To date, 24 MMPs (MMP1, MMP2, MMP3, MMP4, MMP7, MMP8, MMP9, MMP10, MMP11, MMP12, MMP13, MMP14, MMP15, MMP16, MMP17, MMP19, MMP20, MMP21, MMP23a/MMP23b, MMP24, MMP25, MMP26, MMP27, and MMP28) were identified. For MMP1, Sunami et al. found that the expression of MMP1 was significantly correlated with hematogenous metastasis of colorectal cancer, which were further supported by research made by Shiozawa et al. and Bendardaf et al. (12–14) MMP2 and MMP9 comprise the gelatinase subfamily of MMPs. Marcus et al. found that the concentrations of MMP2 protein expression in tumor tissue were significantly higher than that in tumor-free tissue. In addition, the lymph node status was correlated with the expression of MMP2 in plasma, that is, the expression of MMP2 was significantly increased in patients with lymph node metastasis compared with those without (15). MMP7, also known as matrilysin, is frequently overexpressed in human cancer tissues. Adachi et al. found that the expression of MMP7 correlated significantly with the presence of nodal or distant metastases (16, 17). Another member of the gelatinase subfamily, MMP9, was expressed at significantly higher ratios in the sera of persons with CRC compared with normal controls. Overexpression of p38 gamma MAPK was shown to increase MMP9 transcription, enhancing cell invasion (18). Whereas, TGF-β receptor kinase inhibitors can reduce expression of MMP9 and block CRC metastasis to the liver (19, 20). However, for colitis-associated colon cancer, MMP9 has a protective role and acts as a tumor suppressor (21). MMP12, also called metalloelastase, was reported to be associated with both reduced tumor growth and increased overall survival (22). MMP13, sharing structural homology with MMP1, was reported to be associated with advanced cancer stage, and its overexpression can increase the risk of postoperative relapse (23). In addition to the MMPs mentioned above, MMP3, MMP11, and MMP14 were also found to be highly expressed in malignant tumors as compared with normal tissue (24–26).

As previously described, the relationship between MMPs and the prognosis of human CRC was only partly reported. By integrating state-of-art databases, we conducted a systematical analysis for all 24 human MMPs. Differential expression analyses were implemented in public database and our samples. Prognosis analyses were evaluated in The Cancer Genome Atlas (TCGA) and Gene Expression Omnibus (GEO) datasets. Pathway and network analyses were further used to investigate the mechanisms underlying them. To the best of our knowledge, this is among the first bioinformatic analyses to comprehensively evaluate all 24 MMPs in CRC.

## Methods

### Ethics Statement

This study was approved by the Academic Committee of Sun Yat-Sen University, and it was conducted according to the principles expressed in the Declaration of Helsinki.

### Differential Expression Analyses by Oncomine

Oncomine is an online cancer microarray database (https://www.oncomine.org/resource/login.html). Gene expression array datasets from Oncomine were used to analyze the transcription levels of MMPs in different cancers. Differential gene expression analyses of all MMPs were implemented between cancer samples and normal controls. *p*-value was calculated using Student’s *t*-test. Cutoffs of *p*-value and fold change were 0.01 and 1.5, respectively.

### Differential Expression Analyses by GEPIA

Gene Expression Profiling Interactive Analysis (GEPIA) is an interactive web server which was developed by Tang et al. (27) By using a standard processing pipeline, they analyzed the RNA sequencing expression data of 9,736 tumors and 8,587 normal samples. GEPIA provides customizable tumor/normal differential expression analysis, profiling according to cancer types. Cutoff of *p*-value and fold change used in GEPIA were 0.01 and 2, respectively.

### Validation by Quantitative Real-Time Polymerase Chain Reaction

All fresh frozen tissues were archived from The Sixth Affiliated Hospital of Sun Yat-Sen University. The related protocol of human sample usage and the informed consent was approved by the Ethical Review Board of the The Sixth Affiliated Hospital of Sun Yat-Sen University.

Total RNA was extracted from the tumor and normal tissues of 12 patients using Total RNA Kit (Vazyme, China) according to the manufacturer’s instruction. Detailed information of these 12 patients can be found in **Supplementary Table S1**. For cDNA synthesis, 1 μg total RNA was reverse-transcribed into cDNA by Hiscript@ III RT Super Mix with gDNA wiper (Vazyme, Nanjing, China). Quantitative PCR reaction was then performed using 2×SYBR mix (Vazyme, China) and the reaction was run on Applied Biosystems 7500 Real-time PCR system. The Ct values obtained from different samples were compared using the 2-ΔΔCt method. Glyceraldehyde 3-phosphate dehydrogenase (GAPDH) served as internal reference genes. Sequence information of all used primers is listed in **Supplementary Table S2**.

### Protein Levels in UALCAN

UALCAN is a comprehensive, user-friendly, and interactive web resource for analyzing cancer OMICS data. It is built on PERL-CGI with high-quality graphics using JavaScript and CSS (http://ualcan.path.uab.edu/index.html) (28). Using UALCAN, we evaluated the protein level of MMPs in cancer tissue and normal tissue of colorectal cancer patients.

### Protein Level of MMPs in Our Samples

For preparation of protein extracts, 12 pairs of cancer and adjacent normal tissues were crushed with a mortar under ice cold conditions and lysed with RIPA lysis buffer together with protease inhibitors. Cells were collected and lysed with RIPA lysis buffer together with protease inhibitors. After centrifugation at 12,000 rpm at 4°C for 20 min, supernatants were collected and protein concentration was determined using the Pierce™ BCA protein assay (Thermo, Waltham, MA, USA). Proteins were separated by electrophoresis on a 10% SDS-polyacrylamide gel, electroblotted onto a PVDF membrane, and blocked by 5% nonfat dry milk for 1 h. Membranes were then washed in TBST three times for 5 min and then incubated with anti-MMP1 (Abcam, Cambridge, MA, USA), anti-MMP2 (Abcam), anti-MMP3 (Abcam, USA), anti-MMP7 (Abcam, USA), anti-MMP8 (Abcam, USA), anti-MMP9 (Invitrogen, Waltham, MA, USA), anti-MMP11 (Bioss, Beijing, China), anti-MMP12 (Abcam, USA), anti-MMP14 (Abcam, USA), anti-MMP17 (Abcam, USA), anti-MMP19 (Bioss, China), anti-MMP28 (Abcam, USA), anti-Collagen (Abcam), anti-TIMP2 (Bioss, China), or anti-GAPDH (Abcam). Subsequently, the membranes were washed with PBST and incubated for 1 h with goat anti-rabbit IgG (Abcam). Finally, membranes were washed three times and immunoreactivity was determined by using a Chemi DOC™ XRS+ system (BioRad Laboratories, Hercules, CA, USA).

### Clinicopathological and Survival Analyses

By integrating TCGA dataset and standardized survival endpoints defined by Liu et al. recently, we performed clinicopathological and survival analyses (29). Nonparametric Kruskal-Wallis test was used to evaluate the association of American Joint Committee on Cancer (AJCC) stage of colorectal cancer (stage I, stage II, stage III, and stage IV) with the expression of MMPs. Four kinds of survival analyses were implemented, including overall survival (OS), disease-specific survival (DSS), disease-free survival (DFS) also called disease-free interval (DFI), and progression-free survival (PFS) also called progression-free interval (PFI). Disease-free survival is a concept used to describe the period after a successful treatment during which there are no signs and symptoms of the disease that was treated. In addition, by using the GEO dataset GSE39582, we did a relapse-free survival (RFS) analyses (30). As MMP4, MMP23A/MMP23B were not included in the GSE39582 dataset, only 22 MMPs were analyzed in the RFS analyses. Samples were split into two groups by median expression (high vs. low expression), and Kaplan-Meier plot were depicted (denoted with log rank *p*-value). Hazard ratio (HR) and 85% confidence intervals (CIs) were calculated by multivariate Cox regression adjusting the effect of age at diagnosis and sex.

### TCGA Data and cBioPortal

TCGA collected many types of data for each of over 20,000 tumor and normal samples (31). The colorectal cancer dataset, including data from 640 cases with pathology reports, was selected for further analyses of MMPs using cBioPortal (http://www.cbioportal.org/). The genomic profiles included mutations, putative copy number alterations (CNAs) from genomic identification of significant targets in cancer (GISTIC), mRNA expression Z scores (RNA-seq v.2 RSEM), and protein expression Z scores (reversed-phase protein array (RPPA)). Coexpression and network were calculated according to the cBioPortal’s online instructions. By using the expression data in TCGA, we also calculated the correlation of MMPs with each other and several cancer-associated genes, including MYC, TP53, cyclin-D, as well as CDK4/6. The correlation coefficient was calculated using Spearman’s method.

### siRNA Transfection

HCT116 were cultured in Dulbecco’s modified Eagle’s medium (DMEM; Gibco, Waltham, MA, USA) supplemented with 10% fetal bovine serum (FBS; Gibco, USA), penicillin (100 U/ml), and streptomycin (100 μg/ml) at 37°C in a humidified CO_2_ (5%) atmosphere. MMP11, MMP14, MMP17, MMP19, small interfering RNA (siRNA), and nontargeting siRNA (si-control) were purchased from Ribobio (Guangzhou, China) and used at 20 mM. Opti-MEM transfection media and Lipo3000 (Invitrogen) were used to transfect the cells once they reached 50% confluency. Knockdown was assessed by Western blotting after 48 h of transfection. Sequence information of all used primers is listed in **Supplementary Table S3**.

## Results

### Transcriptional Levels of MMPs in Patients With Colorectal Cancer

**Figure 1:**
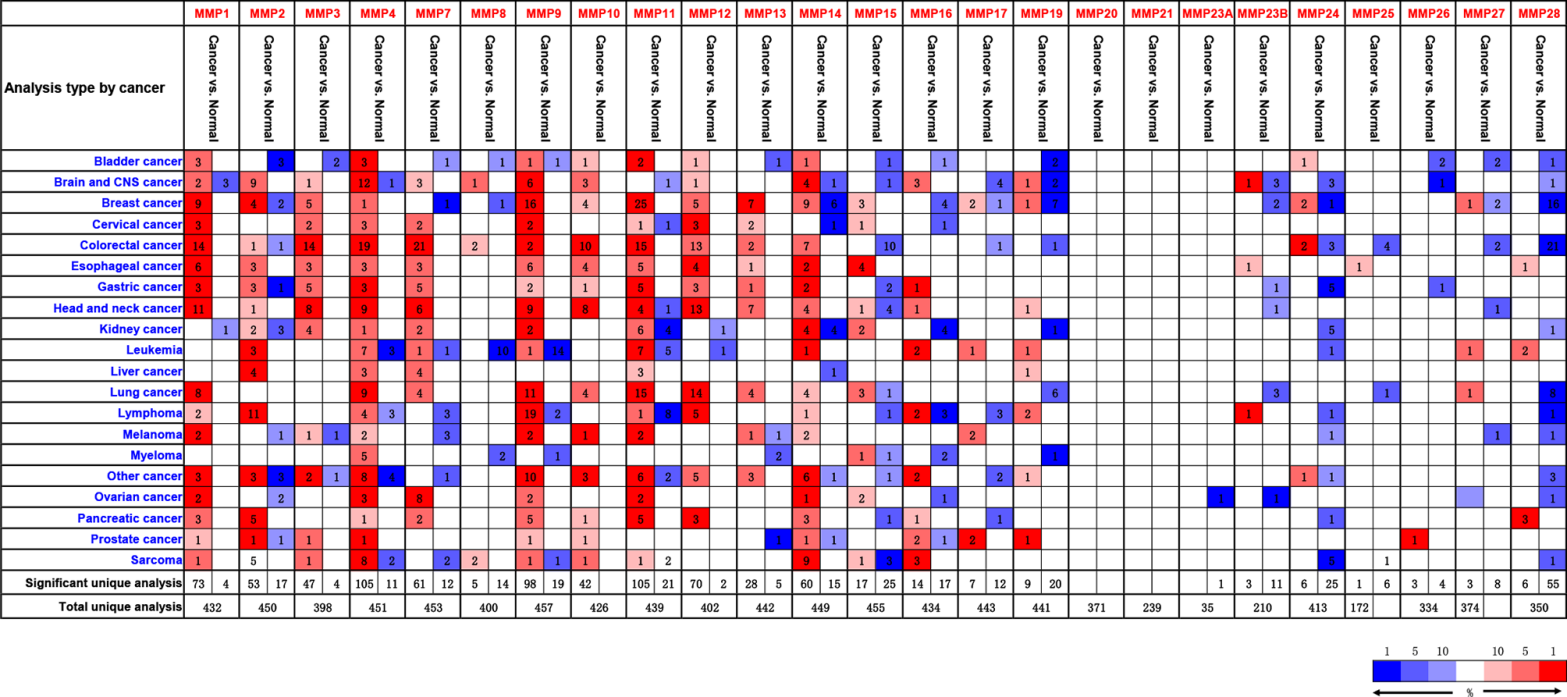
The transcription levels of MMPs in different types of cancers (Oncomine). Upregulated records are highlighted in red, while downregulated records are highlighted in blue. The number in each block means the number of unique analyses, which were fully described in **Supplementary Tables S4**, **S5**.

By using the Oncomine database, we did a Pan-cancer differential gene expression analyses for all MMPs. As shown in **Figure 1**, MMP1–MMP4, MMP7–MMP14, and MMP24 were significantly upregulated in colorectal cancer samples, while MMP15, MMP17, MMP19, and MMP24–MMP28 were significantly downregulated in colorectal cancer samples. Detailed performance of each MMP in Oncomine database can be found in **Supplementary Tables S4**, **S5**.

**Figure 2:**
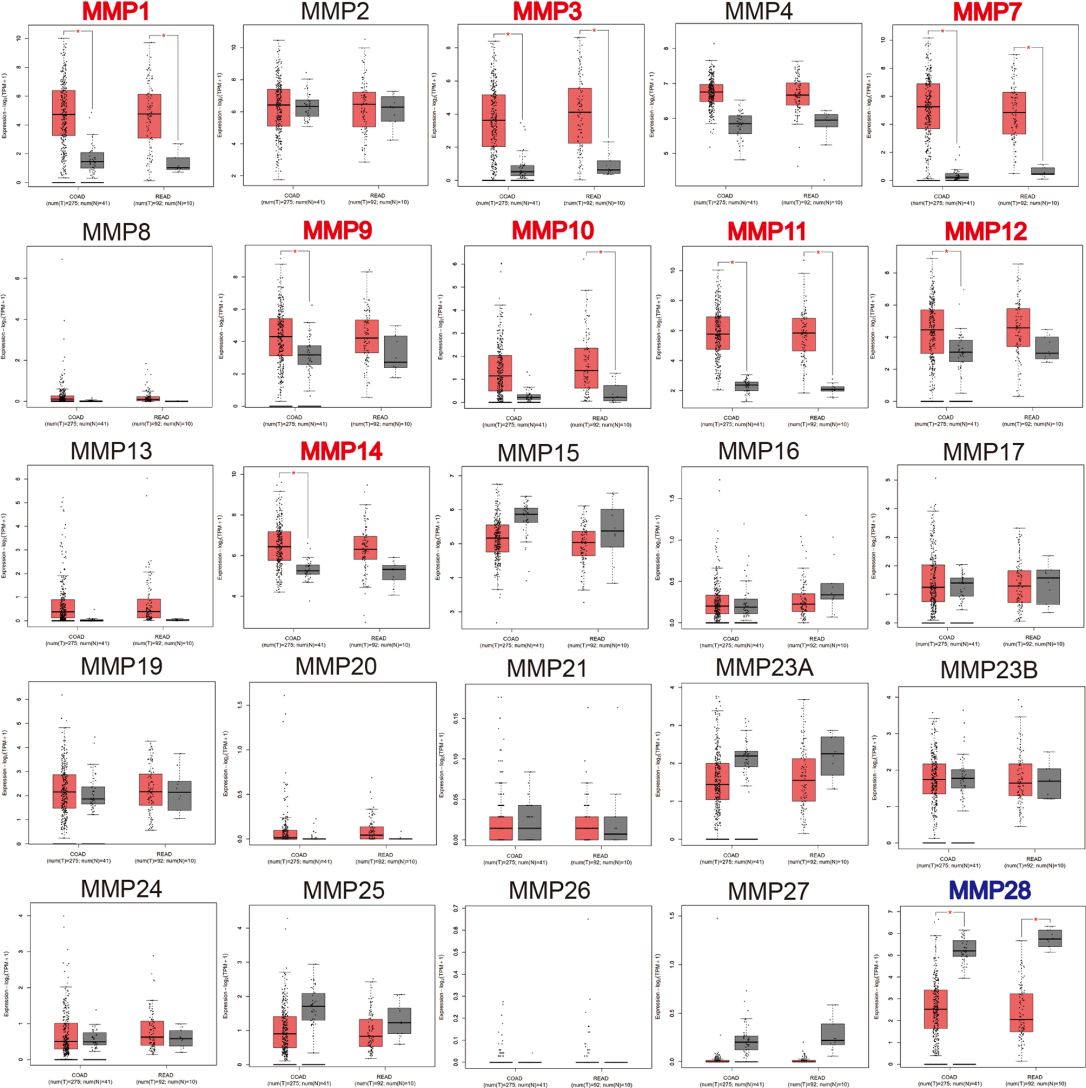
The transcription levels of MMPs in colorectal cancer tissue and normal tissue (GEPIA). Significant records are denoted by the red asterisk on top of the boxplot. The titles of upregulated records are highlighted in red, while the titles of downregulated records are highlighted in blue.

We then used GEPIA to compare the expression level of all MMPs between colorectal tumor tissue and normal tissue. As shown in **Figure 2**, we found that MMP1, MMP3, MMP7, MMP9–MMP12, and MMP14 were significantly upregulated in tumor tissue, while MMP28 was significantly downregulated in tumor tissue.

**Figure 3:**
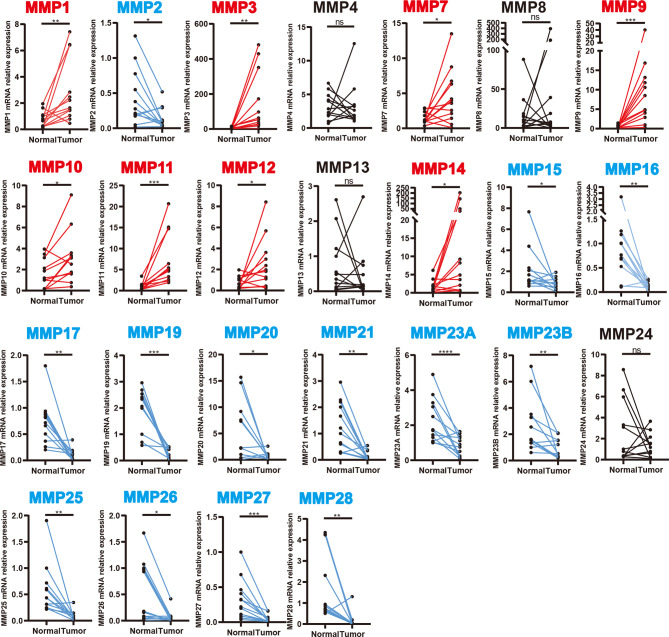
Transcription levels of MMPs in colorectal cancer tissue and adjacent normal tissue (12 colorectal cancer patients in our hospital). Significant records are denoted by the red asterisk on top of the plot (^ns^ *p* > 0.05, ^*^ *p* < 0.05, ^**^ *p* < 0.01, ^***^ *p* < 0.001 and ****p < 0.0001). Upregulated records are highlighted in red, while downregulated records are highlighted in blue.

We further validated the expression level of MMPs in 12 colorectal cancer patients which were recruited from our hospital (including seven patients with colon cancer and five patients with rectal cancer, detailed information can be found in **Supplementary Table S1**) and measured the expression level of 24 MMPs in their tumor tissue and adjacent normal tissue by quantitative real-time polymerase chain reaction (qRT-PCR). As shown in **Figure 3**, we found that MMP1, MMP3, MMP7, MMP9-MMP12, and MMP14 were significantly upregulated in tumor tissue, while MMP15–MMP17, MMP19–MMP21, MMP23A, MMP23B, and MMP25–MMP28 were significantly downregulated in tumor tissue.

In summary, MMP1, MMP3, MMP7, MMP9–MMP12, and MMP14 were consistently upregulated in Oncomine, GEPIA, and our samples. Thus, MMP28 was consistently downregulated in Oncomine, GEPIA, and our samples.

### Protein Levels of MMPs in Patients With Colorectal Cancer

**Figure 4:**
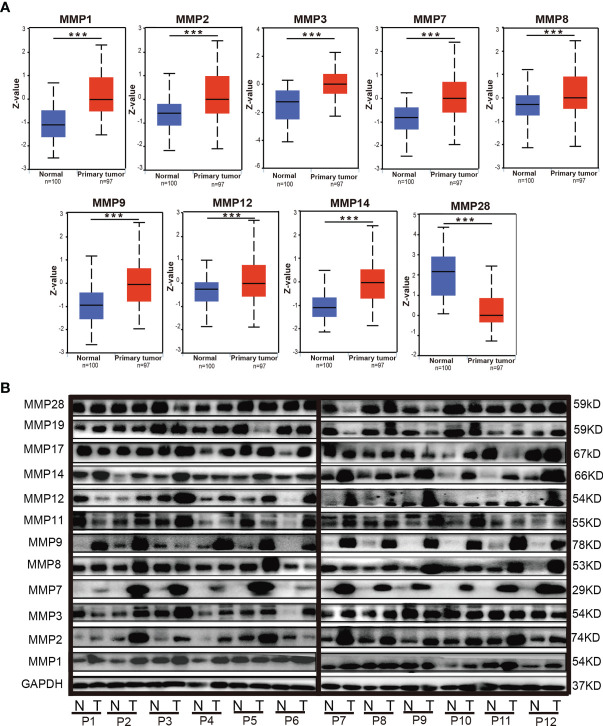
The protein levels of MMPs in colorectal cancer tissue and normal tissue. **(A)** The protein levels of MMPs in UALCAN database; significant records are denoted by the red asterisk on top of the boxplot (^***^ *p* < 0.001). **(B)** The protein levels of MMPs in our 12 samples, which were measured by Western blotting (WB).

By using the UALCAN database, we further evaluated the protein levels of MMPs in patients with colorectal cancer. As some proteins were not included in UALCAN, we can only do the analyses for MMP1, MMP2, MMP3, MMP7, MMP8, MMP9, MMP12, MMP14, and MMP28. As shown in **Figure 4A**, the protein level of MMP1, MMP2, MMP3, MMP7, MMP8, MMP9, MMP12, and MMP14 in colorectal tumor tissue were significantly higher than that in normal tissue, while the protein level of MMP28 in tumor was significantly lower than that in normal tissue.

We also evaluated the protein level of MMPs in our patients and measured the expression level of MMP1–MMP3, MMP7–MMP9, MMP11, MMP12, MMP14, MMP17, MMP19, and MMP28 in their tumor tissue and adjacent normal tissue by Western blot. As shown in **Figure 4B**, we found that the protein level of MMP2, MMP7, MMP9, MMP12, and MMP14 in the tumor tissue were basically higher than that in the normal tissue.

### Relationship Between the mRNA Levels of MMPs and the Clinicopathological Parameters of Patients With Colorectal Cancer

**Figure 5:**
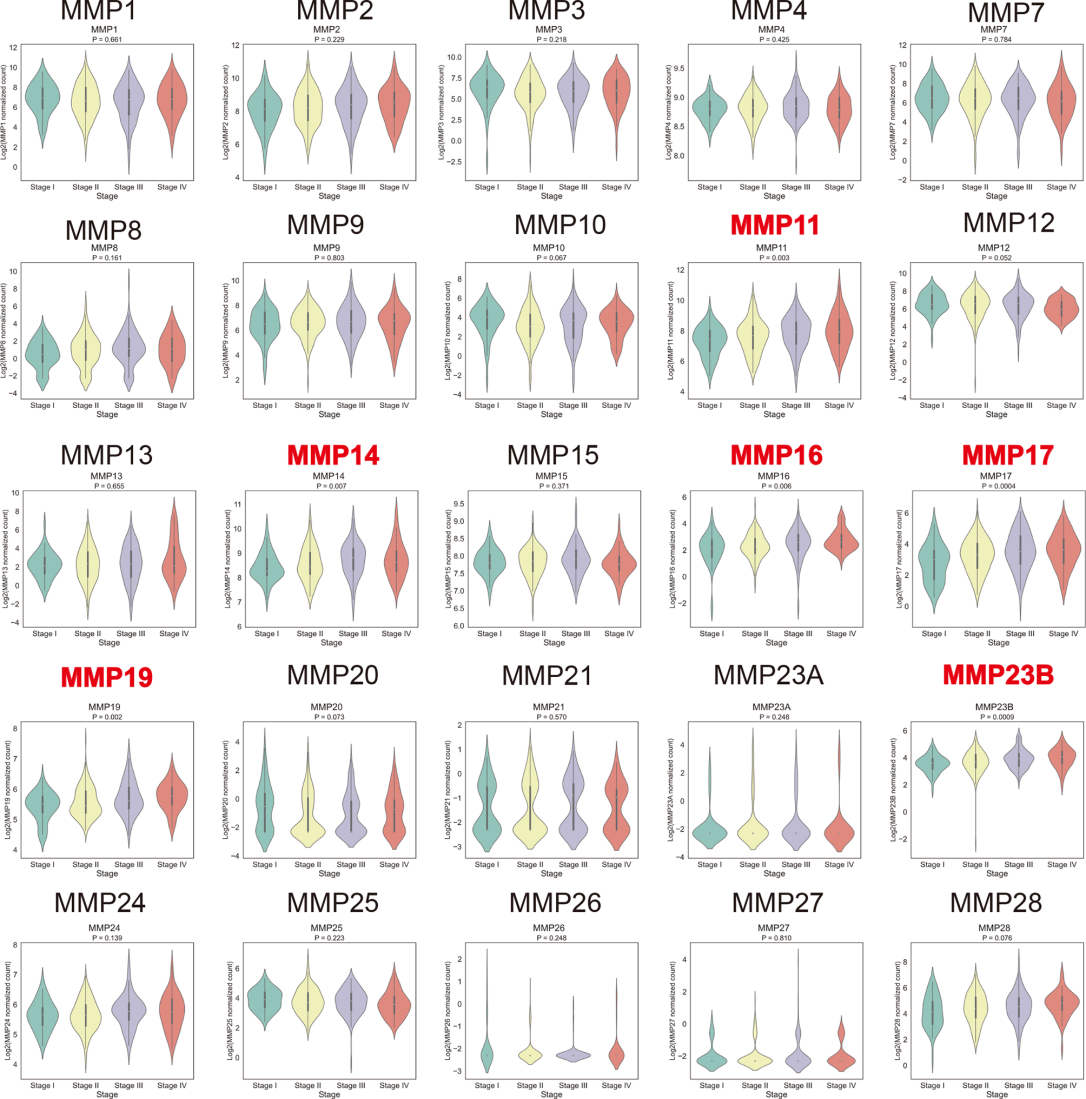
Association of the mRNA levels of MMPs with tumor stage of patients with colorectal cancer. The titles of upregulated records are highlighted in red, while the titles of downregulated records are highlighted in blue.

By using the TCGA dataset, we analyzed the association of MMP expression with the AJCC stage of colorectal cancer. As shown in **Figure 5**, MMP11, MMP14, MMP16, MMP17, MMP19, and MMP23b were positively correlated with the tumor stage, that is, the mRNA levels of MMPs in patients with higher tumor stage were always high. Detailed information can be seen in **Supplementary Table S6**. Take MMP14 as an example, the mean expression level (log2(normalized count of MMP14)) were 8.43, 8.56, 8.74, and 8.72 for stage I, stage II, stage III, and stage IV patients, respectively (*p* = 0.007).

### Association of the mRNA Expression of MMPs With the Prognosis of Patients With Colorectal Cancer

By integrating the TCGA data and four standardized survival endpoints defined by Liu et al. in 2018, we further performed the OS, DSS, DFS, and PFS analyses for all MMPs (**Supplementary Figures S1**–**S4**; **Table 1**). In the OS analyses, upregulated MMP11, MMP16, MMP17, MMP19, and MMP23B were significantly associated with a shorter overall survival time (**Table 1**; **Supplementary Figure S1**); in the DSS analyses, upregulated MMP14, MMP16, MMP17, MMP19, and MMP23B were significantly associated with a shorter disease-specific survival time (**Table 1**; **Supplementary Figure S2**); in the PFS analyses, upregulated MMP11, MMP14, MMP16, MMP17, MMP19, and MMP23B were significantly associated with a shorter progression-free time (**Table 1**; **Supplementary Figure S3**); and in the DFS analyses, downregulated MMP1, MMP3, MMP9, and MMP12 were significantly associated with a shorter disease-free period (**Table 1**; **Supplementary Figure S4**). By using the GEO dataset, we further performed the RFS analyses. As shown in **Supplementary Figure S5** and **Table 1**, upregulated MMP2, MMP11, MMP14, MMP17, MMP19, MMP24, and MMP28 were significantly associated with a shorter relapse-free time, while the downregulated MMP8, MMP13, MMP16, MMP20, and MMP27 was significantly associated with a shorter relapse-free survival time.

**Table 1:** Association of the mRNA expression of MMPs with the prognosis of patients with colorectal cancer.

MMPs	TCGA								GEO	
	Overall survival (OS)		Disease-specific survival (DSS)		Progression-free survival (PFS)		Disease-free survival (DFS)		Relapse-free survival (RFS)	
	HR (95% CI)	*p* ^a^	HR (95% CI)	*p* ^a^	HR (95% CI)	*p* ^a^	HR (95% CI)	*p* ^a^	HR (95% CI)	*p* ^a^
MMP1	0.93 (0.83–1.05)	2.73E−01	0.93 (0.77–1.11)	4.15E−01	0.94 (0.84–1.05)	2.74E−01	**0.69 (0.51–0.92)**	**1.35E−02**	1.03 (0.98–1.09)	2.29E−01
MMP2	1.12 (0.92–1.36)	2.46E−01	1.24 (0.92–1.65)	1.53E−01	1.14 (0.95–1.37)	1.56E−01	0.86 (0.57–1.31)	4.90E−01	**1.17 (1.06–1.29)**	**1.38E−03**
MMP3	0.95 (0.85–1.06)	3.74E−01	0.96 (0.82–1.13)	6.44E−01	0.95 (0.85–1.05)	2.76E−01	**0.72 (0.56–0.93)**	**1.30E−02**	1 (0.94–1.06)	9.62E−01
MMP4	0.94 (0.35–2.49)	8.99E−01	0.87 (0.23–3.22)	8.30E−01	0.63 (0.27–1.49)	2.95E−01	1.07 (0.18–6.43)	9.39E−01	–	–
MMP7	1.11 (0.98–1.27)	9.83E−02	1.17 (0.97–1.41)	1.10E−01	1.06 (0.94–1.18)	3.63E−01	0.97 (0.75–1.25)	8.16E−01	1.09 (1–1.18)	5.36E−02
MMP8	1.13 (0.98–1.31)	1.04E−01	1.09 (0.89–1.33)	4.07E−01	1.12 (0.99–1.27)	7.52E−02	0.97 (0.71–1.33)	8.55E−01	**0.77 (0.64–0.92)**	**4.84E−03**
MMP9	1.05 (0.89–1.24)	5.66E−01	1.05 (0.82–1.33)	7.11E−01	0.97 (0.84–1.14)	7.42E−01	**0.58 (0.37–0.91)**	**1.71E−02**	1.03 (0.93–1.14)	5.35E−01
MMP10	0.91 (0.8–1.03)	1.43E−01	0.86 (0.72–1.04)	1.30E−01	0.93 (0.83–1.05)	2.21E−01	0.9 (0.69–1.17)	4.28E−01	1.02 (0.91–1.14)	7.44E−01
**MMP11**	**1.23 (1.01–1.49)**	**3.71E−02**	1.27 (0.96–1.69)	9.37E−02	**1.33 (1.11–1.59)**	**2.15E−03**	1.27 (0.83–1.94)	2.63E−01	**1.24 (1.09–1.42)**	**1.71E−03**
MMP12	0.96 (0.83–1.11)	5.72E−01	0.91 (0.74–1.13)	3.97E−01	0.94 (0.82–1.07)	3.59E−01	**0.67 (0.48–0.94)**	**2.08E−02**	1.05 (0.99–1.1)	9.10E−02
MMP13	1.04 (0.92–1.17)	5.72E−01	1.08 (0.91–1.28)	3.79E−01	1.03 (0.92–1.15)	6.25E−01	0.76 (0.57–1.01)	5.43E−02	**0.84 (0.73–0.97)**	**1.40E−02**
**MMP14**	1.36 (0.99–1.86)	5.43E−02	**1.73 (1.11–2.68)**	**1.44E−02**	**1.38 (1.04–1.82)**	**2.47E−02**	1.01 (0.54–1.9)	9.69E−01	**1.47 (1.15–1.89)**	**2.48E−03**
MMP15	0.89 (0.53–1.49)	6.53E−01	0.92 (0.44–1.9)	8.14E−01	1.04 (0.66–1.66)	8.56E−01	1.63 (0.59–4.55)	3.49E−01	0.99 (0.85–1.16)	8.95E−01
MMP16	**1.28 (1.02–1.62)**	**3.66E−02**	**1.49 (1.06–2.1)**	**2.08E−02**	**1.25 (1.01–1.56)**	**4.03E−02**	1 (0.59–1.69)	9.94E−01	**0.88 (0.81–0.95)**	**1.97E−03**
**MMP17**	**1.24 (1.04–1.49)**	**1.97E−02**	**1.41 (1.06–1.87)**	**1.84E−02**	**1.19 (1.01–1.41)**	**3.91E−02**	1.36 (0.9–2.06)	1.49E−01	**1.48 (1.14–1.92)**	**3.01E−03**
**MMP19**	**1.92 (1.28–2.88)**	**1.51E−03**	**1.9 (1.08–3.32)**	**2.51E−02**	**1.48 (1.03–2.13)**	**3.47E−02**	1.15 (0.56–2.38)	6.97E−01	**1.31 (1.16–1.49)**	**2.35E−05**
MMP20	1 (0.74–1.37)	9.83E−01	0.87 (0.55–1.38)	5.61E−01	0.94 (0.72–1.23)	6.47E−01	0.65 (0.34–1.23)	1.83E−01	**0.67 (0.51–0.9)**	**6.96E−03**
MMP21	1.44 (0.8–2.58)	2.25E−01	1.19 (0.45–3.12)	7.29E−01	0.9 (0.49–1.66)	7.47E−01	1.37 (0.4–4.71)	6.17E−01	0.54 (0.26–1.12)	9.95E−02
MMP23a	1.12 (0.75–1.69)	5.76E−01	0.86 (0.48–1.56)	6.23E−01	1.01 (0.69–1.48)	9.71E−01	1.59 (0.62–4.07)	3.29E−01	–	−
MMP23b	**1.72 (1.26–2.35)**	**6.96E−04**	**1.64 (1.03–2.59)**	**3.52E−02**	**1.4 (1.06–1.86)**	**1.91E−02**	1.12 (0.59–2.12)	7.24E−01	–	−
MMP24	1.11 (0.72–1.69)	6.43E−01	0.57 (0.31–1.05)	7.28E−02	0.85 (0.58–1.25)	4.19E−01	1.24 (0.46–3.34)	6.75E−01	**1.67 (1.24–2.25)**	**8.48E−04**
MMP25	0.87 (0.68–1.12)	2.91E−01	0.95 (0.66–1.39)	8.08E−01	0.82 (0.65–1.03)	8.39E−02	0.61 (0.34–1.09)	9.51E−02	1.25 (0.98–1.59)	6.65E−02
MMP26	1.4 (0.54–3.67)	4.91E−01	1176.9 (0–0)	1.00E+00	0.61 (0.17–2.14)	4.40E−01	0.02 (0–17787.09)	5.71E−01	1.25 (0.77–2.02)	3.73E−01
MMP27	0.65 (0.2–2.18)	4.89E−01	0.6 (0.08–4.71)	6.29E−01	1.19 (0.71–2)	5.00E−01	0.02 (0–42.44)	3.28E−01	**0.48 (0.3–0.77)**	**2.43E−03**
MMP28	0.97 (0.81–1.16)	7.65E−01	1.05 (0.8–1.37)	7.24E−01	1.09 (0.92–1.28)	3.30E−01	0.94 (0.65–1.37)	7.62E−01	**1.57 (1.19–2.07)**	**1.52E−03**

a Age at diagnosis and sex were adjusted by using multivariate Cox regression. Records with a p<0.05 were bolded. MMP11, MMP14, MMP17 and MMP19 were bolded because they were positively associated with CRC prognosis both in the PFS and in the RFS analyses.

### Prediction Function and Pathways of the Changes in MMPs and Their Frequently Altered Neighbor Genes in Patients With Colorectal Cancer

**Figure 6:**
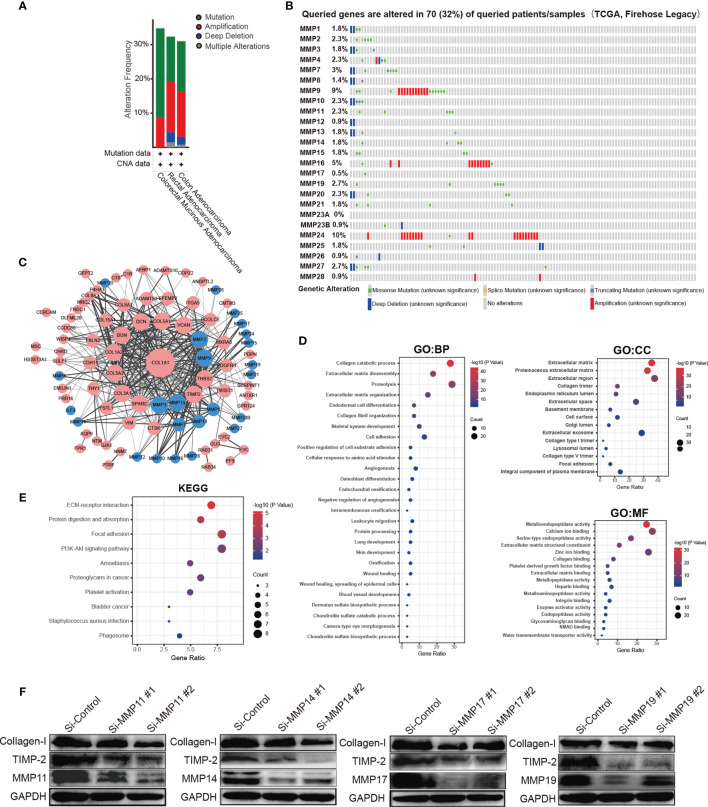
Prediction function and pathways of the changes in MMPs and their frequently altered neighbor genes in patients with colorectal cancer. **(A)** Overview of mutation and copy number validation of MMPs in different types of colorectal cancer, **(B)** detailed alteration proportion and types of each MMP gene, **(C)** network analyses for MMPs and their 50 most frequently altered neighbor genes. **(D)** GO pathway analyses for MMPs and the genes significantly associated with MMP alterations. **(E)** KEGG pathway analyses for MMPs and the genes significantly associated with MMP alterations. **(F)** the expression levels of collagen-I (COL1A1) and TIMP2 after knocking down of MMP11, MMP14, MMP17, and MMP19 in HCT116 cell line by using siRNAs.

We analyzed the MMP alterations and networks by using the cBioPortal online tool for colorectal cancer. As shown in **Figure 6**, of these 220 colorectal cancer patients, MMPs were altered in more than 30% of them (**Figure 6A**). The top 5 altered genes were MMP24 (10%), MMP9 (9%), and MMP16 (5%) (**Figure 6B**). As shown in **Supplementary Figure S6**, we also calculated the correlation of MMPs with each other and several cancer-associated genes, including MYC, TP53, cyclin-D, as well as CDK4/6. We found that multiple MMPs including MMP1, MMP3, MMP4, MMP7, MMP8, and MMP10–MMP14 were positively correlated with the expression of MYC, CCND1, and CDK4/6. We then constructed the network for MMPs and the 80 most frequently altered neighbor genes (**Figure 6C**). The results showed that collagen-related genes (for example, COL1A1) and metalloproteinase inhibitor-related genes (for example, TIMP2) were closely associated with MMP alterations. The functions of MMPs and the genes significantly associated with MMP alterations were predicted by Gene Ontology (GO) and Kyoto Encyclopedia of Genes and Genomes (KEGG) in the Database for Annotation, Visualization and Integrated Discovery (DAVID) (https://david.ncifcrf.gov/summary.jsp).

GO enrichment analyses predicted the functional roles of target host genes on the basis of three aspects, including biological processes, cellular components, and molecular functions. For biological processes, the top 3 pathways were collagen catabolic process, extracellular matrix disassembly, and proteolysis, respectively. For cellular components, the top 3 pathways were extracellular matrix, proteinaceous extracellular matrix, and extracellular region, respectively, and for the molecular functions, the top 3 pathways were metalloendopeptidase activity, calcium ion binding, and serine-type endopeptidase activity, respectively (**Figure 6D**). In the KEGG enrichment analyses, the top 3 pathways were ECM-receptor interaction pathway, protein digestion and absorption pathway, and focal adhesion pathway, respectively (**Figure 6E**). Finally, by knocking down the expression of MMP11, MMP14, MMP17, and MMP19, we found that the expression of TIMP2 were significantly downregulated (**Figure 6F**). Similar trends were found for collagen-I (COL1A1) but not so obvious as TIMP2.

## Discussion

MMPs were reported to be associated with the progression of colorectal cancer; however, a comprehensive bioinformatic analysis for all MMPs has yet to be performed. In this study, we systematically explored the mRNA expression level of all 24 MMPs and their prognosis value in colorectal cancer. We found that, the transcriptional level of MMP1, MMP3, MMP7, MMP9–MMP12, and MMP14 in tumor were significantly upregulated, both in public database and in our samples. Also, in the clinicopathological and prognosis analyses, upregulated MMP11, MMP14, MMP17, and MMP19 were significantly associated with a higher tumor stage and a worse prognosis.

In this study, five survival endpoints were used in the survival analyses. OS is an important endpoint and is easy to define (the patient is either alive or dead). However, using OS as an endpoint may weaken a clinical study as deaths because of noncancer causes that do not necessarily reflect tumor biology. DSS can overcome the shortage of OS as DSS only considers the people who have not died from a specific disease in a defined period of time. However, both OS and DSS demand longer follow-up times; thus, in many clinical trials, DFS or PFS are preferred. PFS is defined as the time to disease progression or death from any cause. Whereas, DFS is used to describe the period after a successful treatment during which there are no signs and symptoms of the disease that was treated. The above four endpoints of TCGA dataset were standardized by Liu et al. in 2018 (29). Another survival endpoint, RFS which was used by the GEO database (GSE39582), was defined as the time from surgery to the first relapse and was censored at 5 years (30).

MMP11 also named stromelysin-3 is a member of the stromelysin subgroup belonging to the MMP superfamily. In this study, MMP11 was significantly upregulated in tumor, both in public database (Oncomine and GEPIA) and in our samples. The protein levels of MMP11 in our 12 pair samples were upregulated in tumor tissue for patients 2, 3, 6, 9, and 10 but not for other patients. Also, in the clinicopathological and survival analyses, upregulated MMP11 was significantly associated with a higher tumor stage (*p* = 0.003), a shorter OS (HR = 1.23, *p* = 3.71 × 10^−2^), a shorter PFS time (HR = 1.33, *p* = 2.15 × 10^−3^), and a shorter RFS time (HR = 1.24, *p* = 1.71 × 10^−3^). In the DSS and DFS analyses, although the association did not reach a significant level, a similar trend was found (HR >1). In a previous study, Li et al. measured the serum levels of MMP11 in 92 colon cancer patients and 92 healthy individuals using ELISA. They found that the serum levels of MMP11 were substantially higher in colon cancer patients than in healthy controls and was an independent predictor of the OS and DFS of colon cancer (32). MMP11 also played an important role in the tumorigenesis, proliferation, and invasion process of other cancers (33, 34). The mechanism behind it, may by inhibiting apoptosis as well as enhancing migration and invasion of cancer cells (35).

MMP14 plays an important role in extracellular matrix remodeling during aging. It has been reported to interact with TIMP2 (36). In our network analyses (**Figure 6C**), TIMP2 was indeed the closest gene of MMP14. Thus, by knocking down MMP14, the expression of TIMP2 was significantly downregulated (**Figure 6F**). In the transcriptional level, MMP14 was significantly upregulated in tumor tissue both in the public database and our own subjects. In the protein level, MMP14 was significantly upregulated in tumor tissue both in the UALCAN database and in patients 2, 3, 7, 9, 10, 11, and 12 of our own subjects. Furthermore, in the clinicopathological and prognosis analyses, upregulated MMP14 was significantly associated with a higher tumor stage (*p* = 0.007), a shorter DSS survival time (HR = 1.73, *p* = 0.01), a shorter PFS time (HR = 1.38, *p* = 0.02), and a significantly shorter RFS time (HR = 1.47, *p* = 2.48 × 10^−3^). The association of MMP14 with the prognosis of colorectal cancer was also reported by Cui et al. in 2019. In addition, Cui et al. found that patient with upregulated MMP14 was significantly associated with a lower 5-year DFS and OS (37). Recently, Ragusa and coworkers found that upregulated MMP14 levels correlated with blood vessel dysfunction and a lack of cytotoxic T cells (38).

MMP17 and MMP19 were another two MMPs. In this study, we found that upregulated MMP17 and MMP19 were significantly associated with a higher tumor stage (*p* = 4 × 10^−4^ and *p* = 2 × 10^−3^ for MMP17 and MMP19, respectively), a shorter OS time (HR = 1.24, *p* = 0.02 for MMP17 and HR = 1.92, *p* = 1.51 × 10^−3^ for MMP19), a shorter DSS time (HR = 1.41, *p* = 0.02 for MMP17 and HR = 1.9, *p* = 0.03 for MMP19), a shorter PFS (HR = 1.19, *p* = 0.04 for MMP17 and HR = 1.48, *p* = 0.03 for MMP19), and a shorter RFS (HR = 1.48, *p* = 3.01 × 10^−3^ for MMP17 and HR = 1.31, *p* = 2.35 × 10^−5^ for MMP19). However, in the transcriptional analyses, MMP17 and MMP19 were significantly upregulated in Oncomine and our 12 samples but not in the GEPIA. In the protein analyses of our samples, MMP17 was upregulated in tumor tissue for patients 4, 6, and 10. Recently, by detecting MMP19 mRNA expression in 198 CRC cancer tissues and paired normal controls, Chen et al. found that MMP19 expression was significantly upregulated in cancer tissues than in normal controls. In addition, by using immunohistochemistry to detect the expression of MMP19 protein in 42 patients, they further found that MMP19 mRNA expression is highly correlated with their protein levels. In their prognosis analyses, significant association between upregulated MMP19 expression and worse prognosis was also found (39). The transcriptional level of MMP17 and MMP19 in colorectal cancer tissue and normal tissue may need to be further confirmed.

Recently, a Pan-cancer analysis for MMPs in TCGA was implemented by Emily et al. (40) Different from our study, they focus on the overall performance of MMPs in several cancers. In their study, they only used the colon cancer patients (COPD) of TCGA (without rectal cancer) and there is no validation dataset. In addition, the only survival endpoint used in their analyses was OS. As we described above, due to shorter follow-up time in TCGA, the accuracy of OS may not be as good as PFS. Finally, without adjusting the effect of age at diagnosis and sex, the log-rank test used in their study may bias the final result.

In summary, our study was among the first study to systematically evaluate the performance of MMPs in colorectal cancer. This study will deepen our understanding of the prognosis mechanism of colorectal cancer. Also, MMP11, MMP14, MMP17, and MMP19 are potential targets of precision therapy for patients with colorectal cancer.

## Data Availability Statement

The original contributions presented in the study are included in the article/**Supplementary Material**. Further inquiries can be directed to the corresponding authors.

## Ethics Statement

The studies involving human participants were reviewed and approved by the Academic Committee of Sun Yat-Sen University. The patients/participants provided their written informed consent to participate in this study.

## Author Contributions

JY: acquisition of data, statistical analysis and technical interpretation of data, drafting of the manuscript, and critical revision of the manuscript for important intellectual content. ZH: acquisition of data and material, technical, and administrative support. XH, ZL, and LL: acquisition of data or material support. PL: study concept and design, acquisition of data, material support, analysis and interpretation of data, critical revision of the manuscript for important intellectual content, and administrative support. HC: study concept and design, analysis and interpretation of data, critical revision of the manuscript for important intellectual content, and administrative support. All authors contributed to the article and approved the submitted version.

## Funding

This study was supported by the National Key R&D Program of China (PL, 2017YFC1308800); National Natural Science Foundation of China (PL, 81970452; ZH, 81902938); Science and Technology Program of Shenzhen, China (PL, JCYJ20190807161807867); Natural Science Foundation of Guangdong Province, China (ZH, 2020A1515011248); Fundamental Research Funds for the Central Universities (ZH, 19ykzd02); Guangdong Science and Technology Department (HC and BW, 2020B1212060018); the National Natural Science Foundation of China (HC, 81802833); and the 100 Top Talent Programs of Sun Yat-Sen University (HC, 58000-18841290).

## Conflict of Interest

The authors declare that the research was conducted in the absence of any commercial or financial relationships that could be construed as a potential conflict of interest.

## Publisher’s Note

All claims expressed in this article are solely those of the authors and do not necessarily represent those of their affiliated organizations, or those of the publisher, the editors and the reviewers. Any product that may be evaluated in this article, or claim that may be made by its manufacturer, is not guaranteed or endorsed by the publisher.
