# Supplementary figures and images for: Comprehensive Analysis of the Expression and Prognosis for MMPs in Human Colorectal Cancer
Source: Front Oncol. 2021 Nov 5;11:771099. doi: 10.3389/fonc.2021.771099 (PMC8602079; doi:10.3389/fonc.2021.771099)

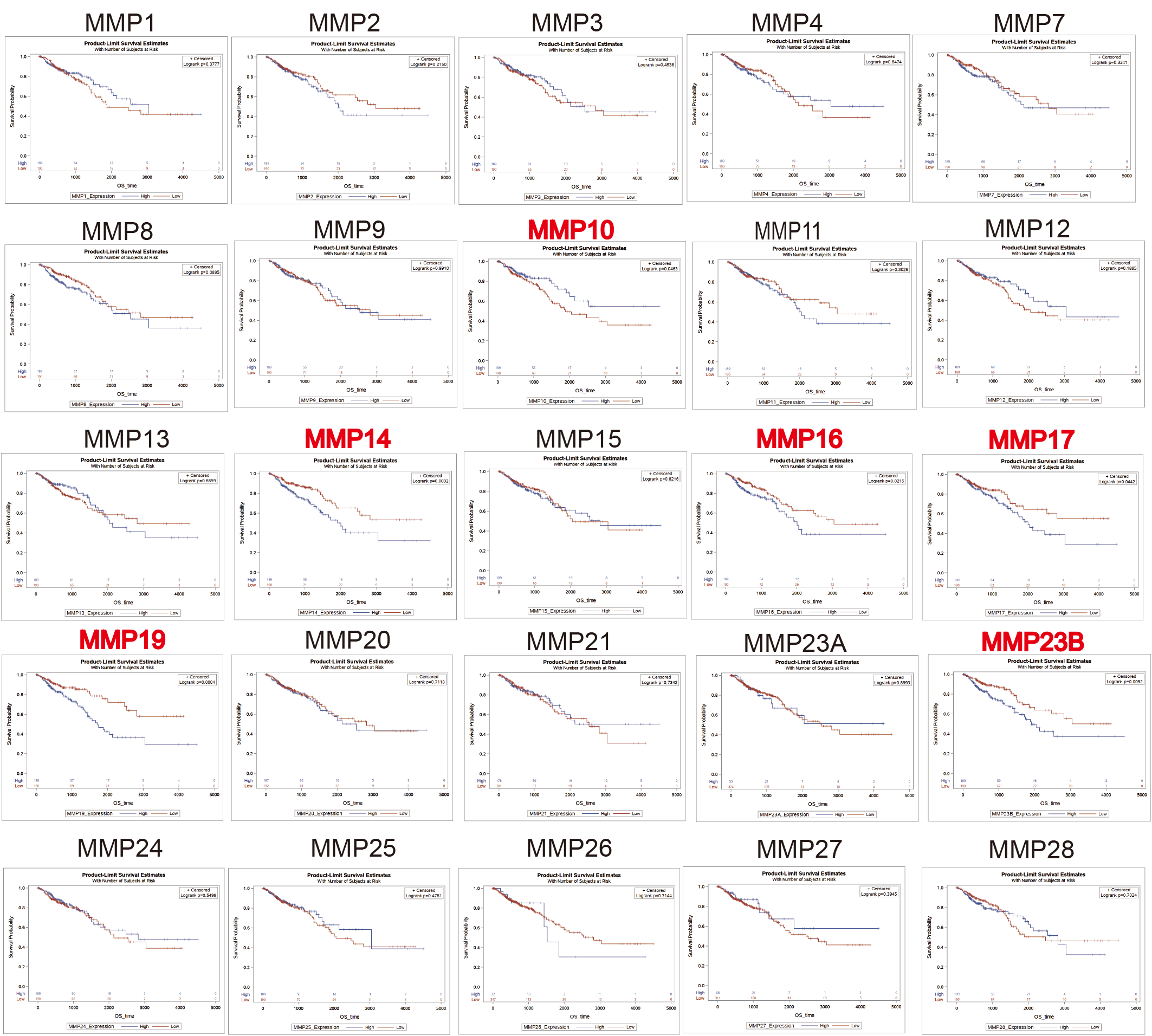

Supplement: Supplementary file 1 [file Image_1.tif]

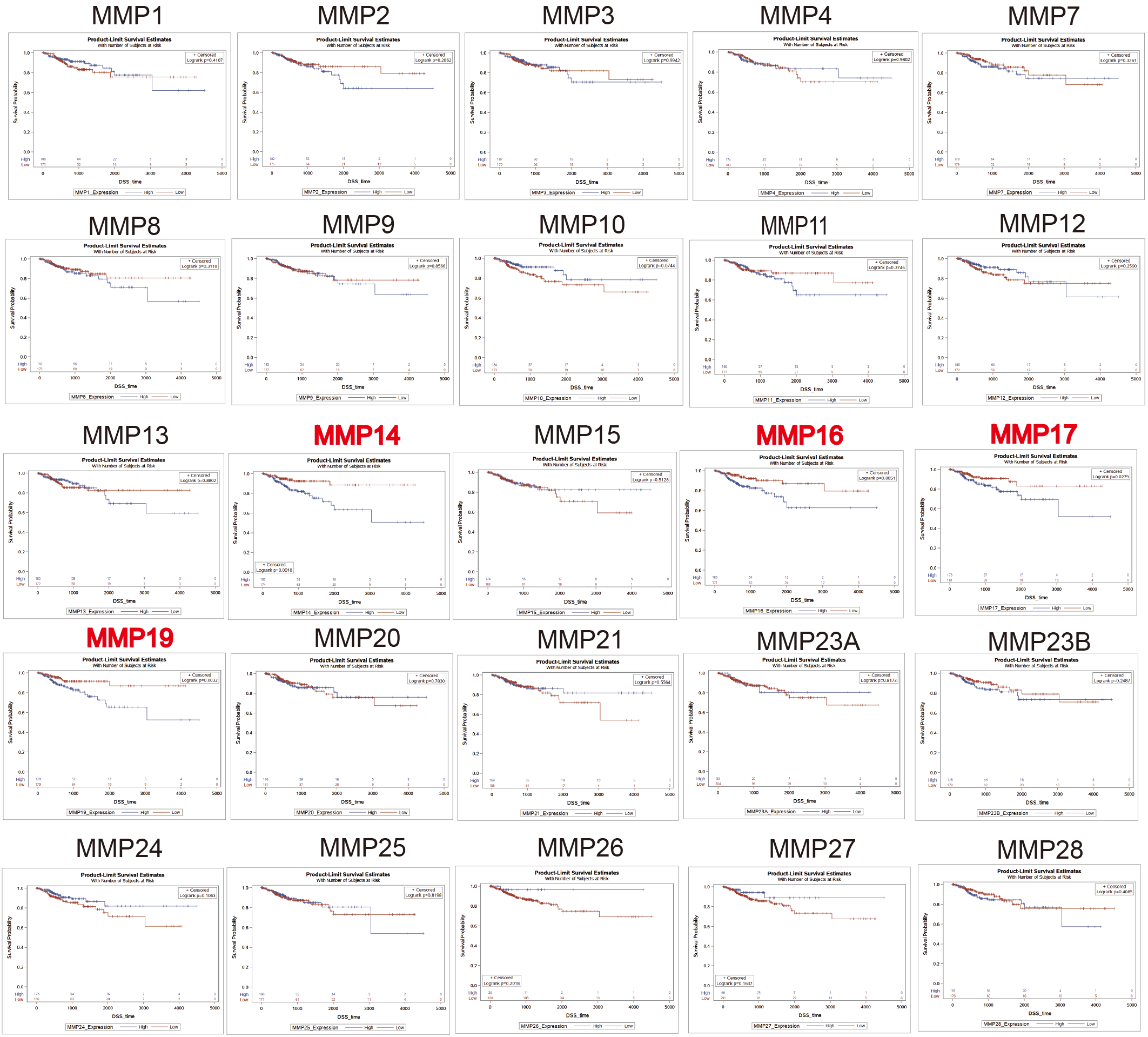

Supplement: Supplementary file 2 [file Image_2.tif]

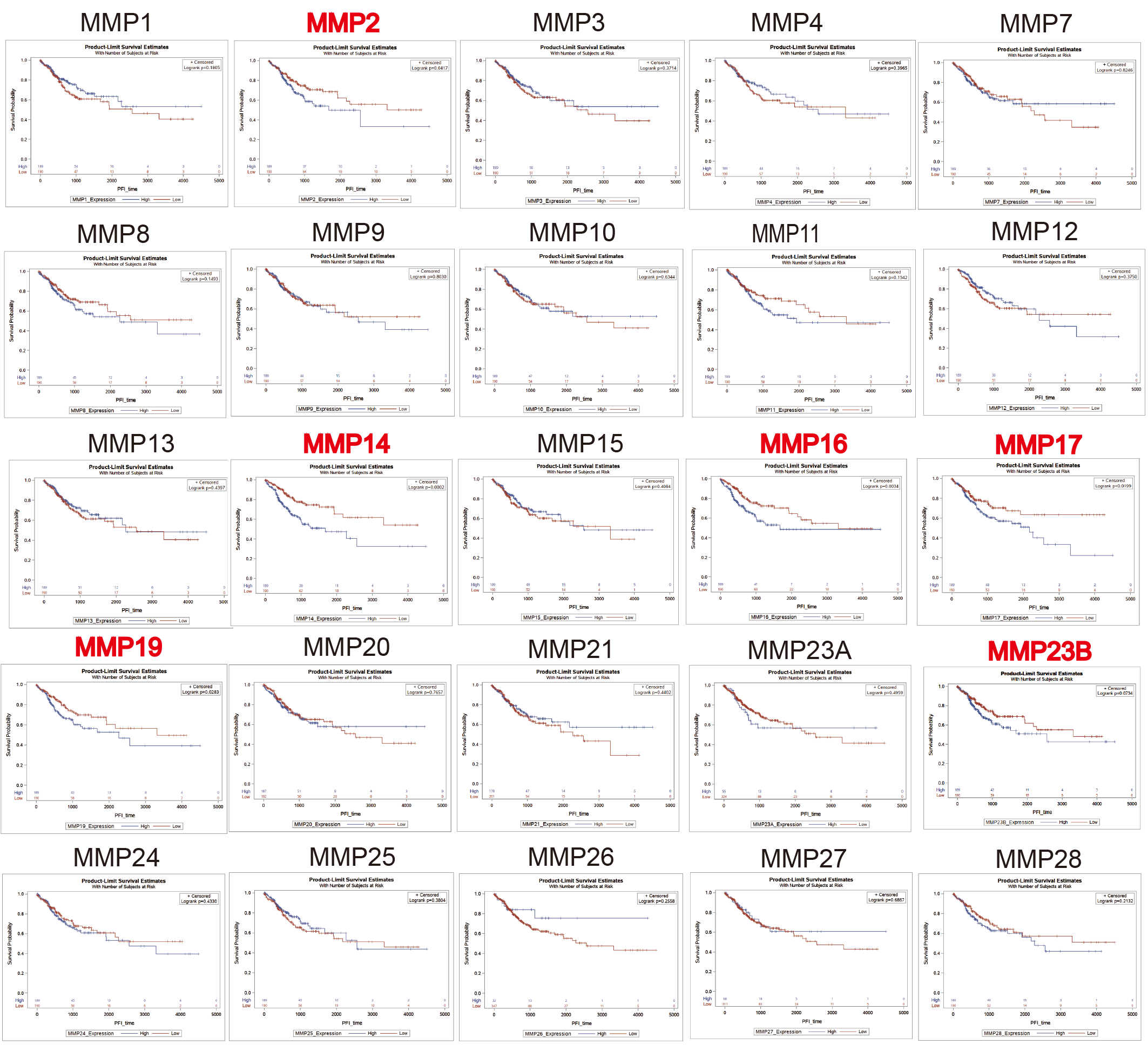

Supplement: Supplementary file 3 [file Image_3.tif]

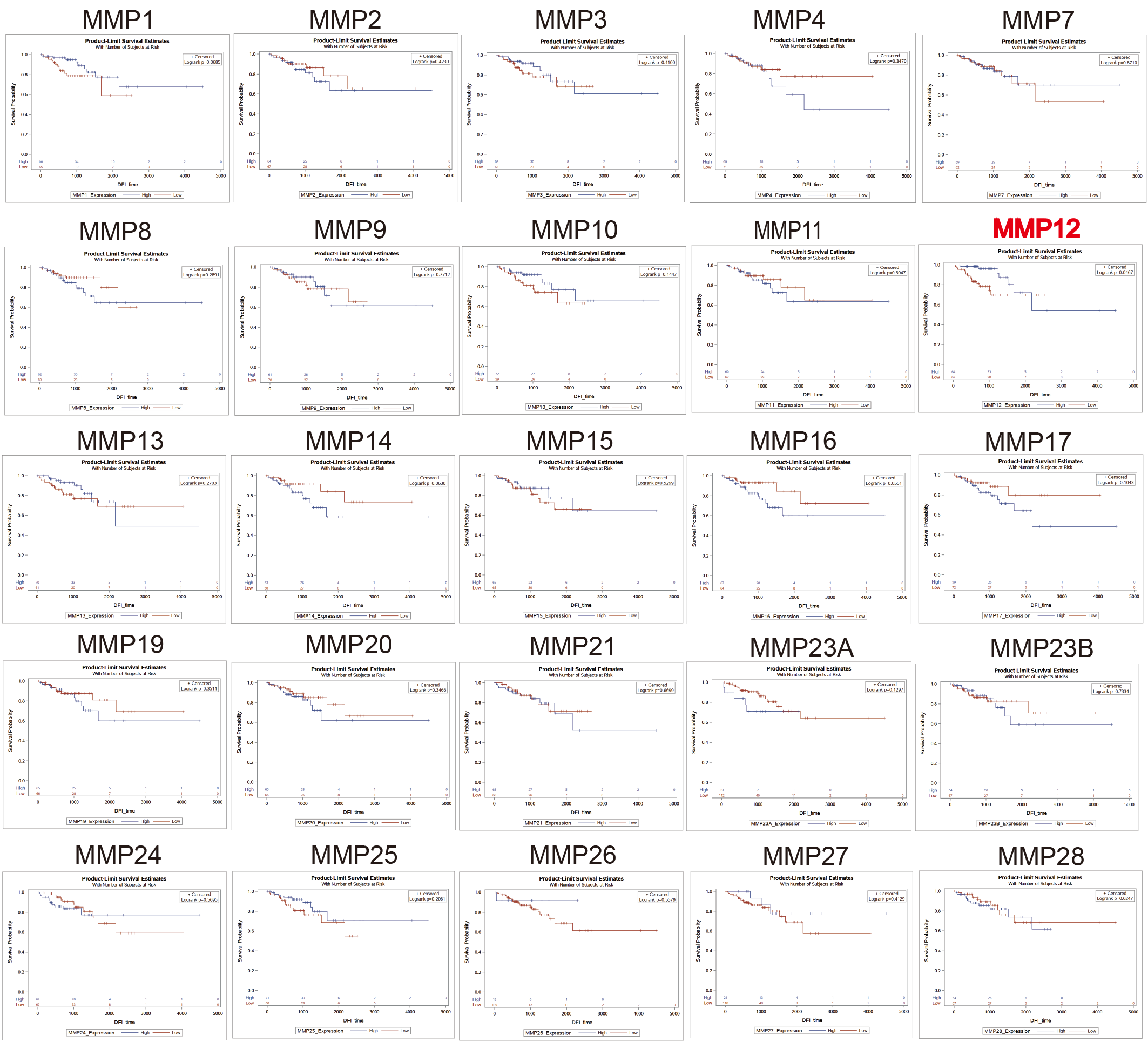

Supplement: Supplementary file 4 [file Image_4.tif]

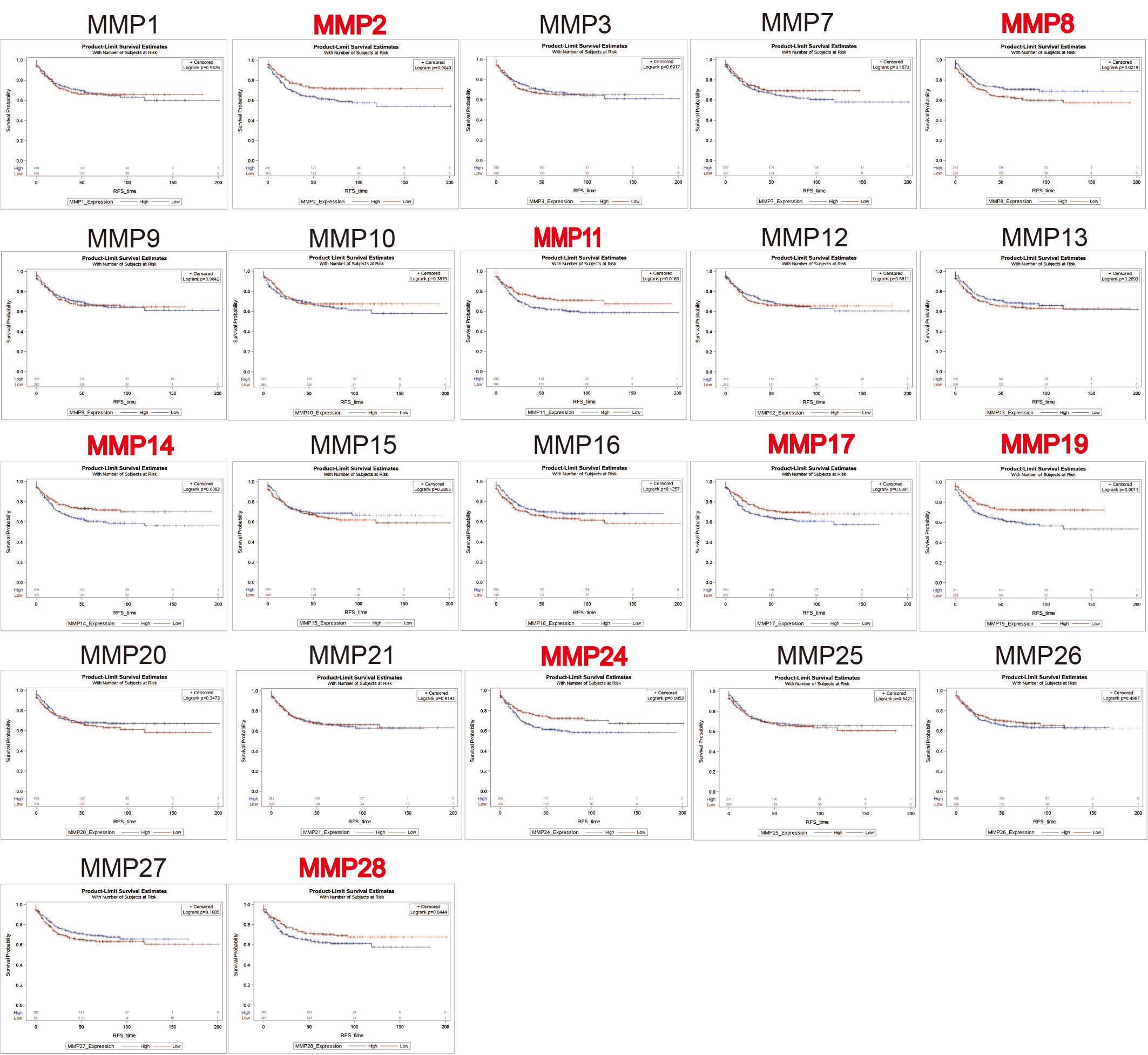

Supplement: Supplementary file 5 [file Image_5.tif]

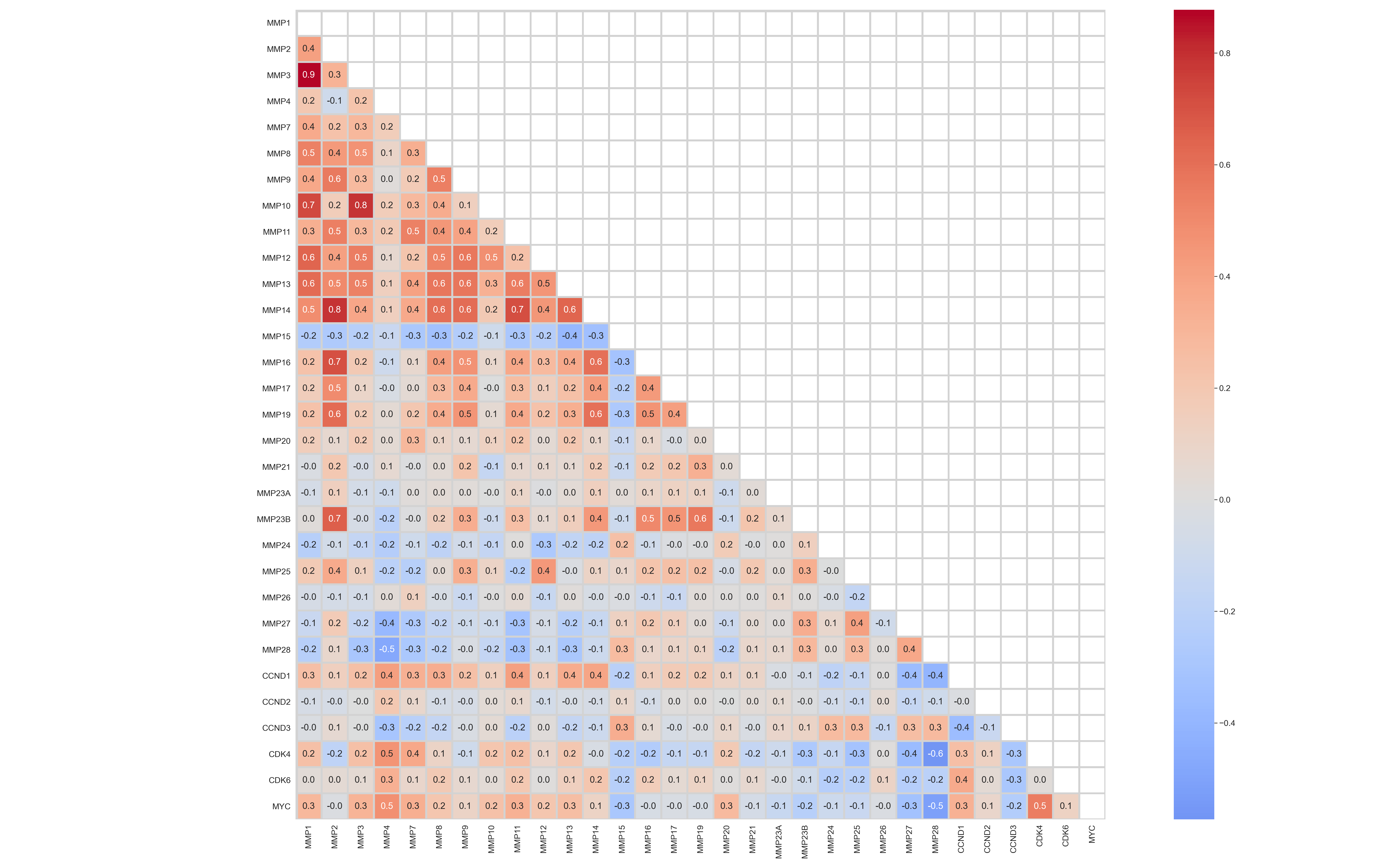

Supplement: Supplementary file 6 [file Image_6.tif]
